# Changes in event-related potentials in patients with first-episode schizophrenia and their siblings

**DOI:** 10.1186/s12888-016-1189-7

**Published:** 2017-01-17

**Authors:** Chengqing Yang, Tianhong Zhang, Zezhi Li, Anisha Heeramun-Aubeeluck, Na Liu, Nan Huang, Jie Zhang, Leiying He, Hui Li, Yingying Tang, Fazhan Chen, Jijun Wang, Zheng Lu

**Affiliations:** 1 Department of Psychiatry, Shanghai Mental Health Center, Shanghai Jiao Tong University School of Medicine, 600 Wan Ping Nan Road, Shanghai, 200030 China; 2 Department of Psychiatry, Tongji Hospital, Tongji University School of Medicine, 389 Xin Cun Road, Shanghai, 200065 China; 3 Department of Neurology, Renji Hospital, Shanghai Jiaotong University School of Medicine, 160 Pujian Road, Shanghai, 200127 China

**Keywords:** First-episode schizophrenia, Event-related potential, Genetic high-risk population

## Abstract

**Background:**

This study aimed to explore the characteristics of event-related potentials induced by facial emotion recognition in patients with first-episode schizophrenia and in their siblings.

**Methods:**

In this case-control study, 30 first-episode schizophrenia patients, 26 siblings, and 30 healthy controls were enrolled. They completed facial emotion recognition tasks from the Ekman Standard Faces Database as an induction for evoked potentials. Evoked potential data were obtained using a 64-channel electroencephalography system. Average evoked potential waveforms were computed from epochs for each stimulus type. The amplitudes and latency of the event-related potentials for P100 (positive potential 100 ms after stimulus onset), N170 (negative potential 170 ms after stimulus onset), and N250 (fronto-central peak) were investigated at O1, O2, P7, and P8 electrode locations.

**Results:**

There were significant differences between the groups for P100 amplitude (F = 11.526, *P* < 0.001), electrode position (F = 450.592, *P* < 0.001), emotion (disgust vs. happiness vs. fear) (F = 1722.467, *P* < 0.001), and emotion intensity (low vs. moderate vs. high) (F = 1737.169, *P* < 0.001). Post hoc analysis showed significantly larger amplitudes in the schizophrenia group at the O1, O2, P7, and P8 electrode positions. There were no significant differences between the siblings of schizophrenia patients and the healthy controls.

**Conclusions:**

Patients with schizophrenia showed abnormalities in P100 amplitude, but similar results were not observed in their siblings. These results provide evidence of dysfunctional event-related potential patterns underlying facial emotion processing in patients with schizophrenia. P100 may be a characteristic index of schizophrenia.

**Electronic supplementary material:**

The online version of this article (doi:10.1186/s12888-016-1189-7) contains supplementary material, which is available to authorized users.

## Background

Basic facial emotions include happiness, anger, sadness, fear, surprise, and disgust, each having its own distinct facial expression. The recognition of emotional expressions is pivotal to effective communications and to social functioning and quality of life. Impairments in facial emotion recognition and social cognition that underlies social interactions, which include emotion processing, theory of mind (ToM), and social relationship perception, have been consistently shown in patients with chronic schizophrenia [1–4]. Patients with schizophrenia not only show general deficits in emotion recognition, but also impairments in processing negative emotions that appear to be greater than those for positive ones such as happiness [5–7]. Whether these impairments are core features of schizophrenia or are consequent to its chronicity or treatments remain controversial because of the paucity of data about the deficits at the first episode of schizophrenia [1]. Evidence suggesting that deficits in emotion recognition are present in early stages of the disease could improve our understanding of the pathophysiology of schizophrenia, which could suggest potential targets for treatment [8].

Over the past decade, studies of emotion processing have shifted their focus from schizophrenic patients to persons at risk of the disease, in order to try to determine whether emotion perception abilities represent an endophenotypic marker related to the risk of schizophrenia [9]. Siblings of patients diagnosed with schizophrenia have an increased risk of schizophrenia compared to healthy controls [10], but few studies have examined whether they also exhibit differences in facial emotion recognition deficits. Siblings of patients with schizophrenia may have some degree of emotion perception impairment, but these impairments could be less important than in patients with schizophrenia. Therefore, differences between siblings and control populations may be hard to detect using conventional methods [11, 12].

We recently demonstrated that deficits in facial emotion recognition were related to executive function impairment in patients with schizophrenia as well as in their siblings [13]. We wanted to estimate the extent of facial emotion recognition deficits in both patients with schizophrenia and their siblings and to determine whether the impairments in siblings not diagnosed with schizophrenia could be identified using event-related potential (ERP). The use of ERP waveforms to measure neural activity during emotion processing has become a major approach in cognitive affective neuroscience. This method captures the exact time course of the emotional information-processing cascade, from the early to the late processing stages with a millisecond-resolution [14], each component distinctly shown as negative and positive deflections. Early sensory processing of visual stimuli is recorded by posterior occipital electrodes and shown as a positive potential about 100 ms after stimulus onset (P100) [15]. An impaired P100 component was found in patients with schizophrenia, suggesting that schizophrenia may be associated with impairment of early dorsal visual stream processing [16]. Early stages of information processing of the face, including the analysis of its structure and the configuration of its elements [17], is measured by the N170 component (negative potential 170 ms after stimulus). The N170 component is generated in the fusiform gyrus, which is believed to be insensitive to emotional expression [18]. The N250 peak (sometimes also defined as the N240 component) is generated at fronto-central sites and has been related to the recognition of complex aspects of facial information such as emotional content and identity, associating the face with semantic and cognitive information [19].

In the current study, the P100, N170, and N250 components were measured and compared among first-episode schizophrenia patients, their siblings, and healthy controls.

## Methods

### Participants

The study population has been previously described [13]. This was a case-control study of first-episode schizophrenia patients hospitalized at the Early Assessment Service for Young People with Psychosis (EASY) of the Shanghai Mental Health Center between May 2008 and May 2012. All included participants were diagnosed with schizophrenia according to the Statistical Manual of Mental Disorders, 4th edition (DSM-IV). These patients had not been subjected to electroconvulsive treatment during the past 3 months and their duration of illness was <3 years, not including the prodromal phase.

Since the simple fact of being a sibling of a patient with schizophrenia increases the risk of schizophrenia [13], siblings of the enrolled patients with schizophrenia were recruited and matched for age, gender, and education with the patients. Both participants with schizophrenia and their at-risk siblings presenting any of the following conditions were excluded: history of alcohol and drug abuse, head injury, neurological disorder, dementia, any Axis I psychiatric disorders, or mental retardation.

Thirty healthy controls were recruited from community volunteers and hospital staff. They were matched for age, gender, and education to the patients with schizophrenia. We used the DSM-IV axis I disorder Structured Clinical Interview for Diagnostic (SCID assessment form) to exclude a history of mental disorders and family history of mental disorders. The above exclusion criteria were also applied to the control participants.

The study was approved by the ethics committee of the Shanghai Mental Health Center affiliated to Jiao tong University. All subjects provided a written informed consent. This study adhered to STROBE guidelines/methodology. The demographic data of all subjects were collected. Clinical symptoms were assessed using the Positive and Negative Symptom Scale (PANSS) [20]. Assessment was carried out by two experienced psychiatrists at the Shanghai Mental Health Center (Kappa was 0.87).

### Electroencephalography (EEG) recording and preprocessing

A facial emotion recognition task based on face photographs obtained from the Ekman Standard Face Database, was used as stimulus for ERP, as previously described [13]. Three facial emotions (happiness, fear, and disgust) at three different intensities (low, moderate, and high) were presented four times to each participant; thus, each subject was required to recognize 36 faces in total. Each facial expression was presented for 400 ms and blacked for 1600 ms, during which time the subjects were instructed to recognize the emotion as soon as possible.

The electrode caps had an equidistant layout and covered the whole head. Electrooculography (EOG) electrodes were placed below the left and above the right external canthi using bipolar electrodes recording horizontal and vertical eye movements. The electrodes were applied to the scalp using a 64-channel Quickcap (Brain Product) in an expanded 10–20 system scalp montage referenced to the left mastoid. Electrical potentials were amplified with a Neuroscan Synamps 64-channel amplifier (gain: 1000; range: 5.5 mV; bandpass filter settings: 0.10-50.0 Hz). Data were stored for off-line analysis. The continuous electroencephalography (EEG) was visually scanned for gross artifacts and digitally filtered with a 0.50-30.0 Hz zero phase-shift bandpass filter (12 dB/octave). An eye-movement correction algorithm with established reliability and validity was applied to reduce EOG contamination [21, 22].

**Fig. 1:**
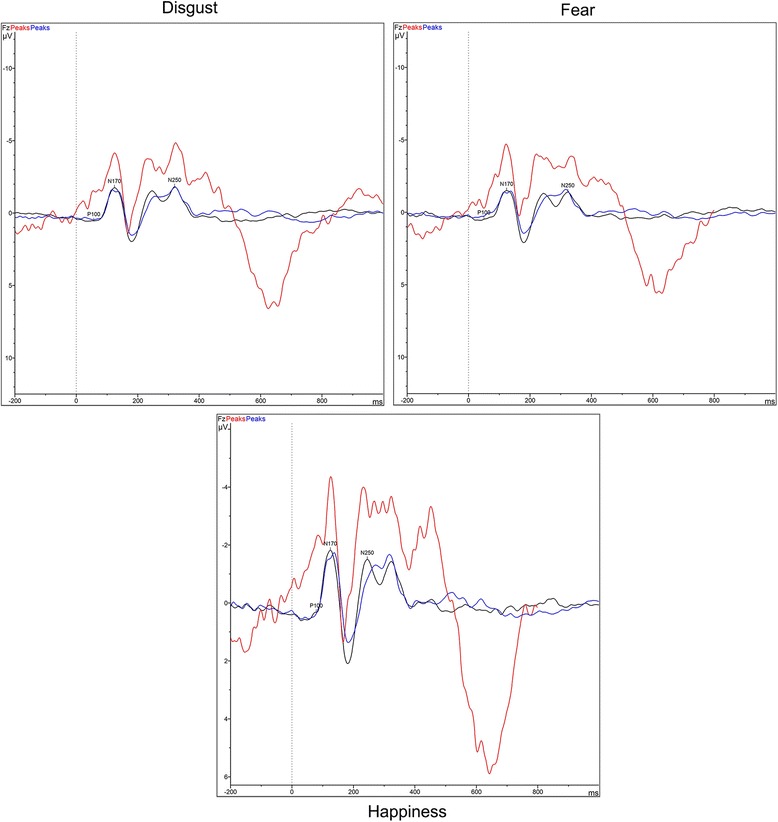
Average waveforms obtained following disgust/fear/happiness recognition in patients with schizophrenia, siblings and healthy controls. (*blue* = patients, *red* = siblings, *black* = control participants)

The continuous recordings were segmented into epochs extending from 100 ms pre-stimulus to 900 ms post-stimulus and re-referenced to the mathematical average of all scalp potentials at each time point, in order to provide a set of unbiased measures. Average evoked potential waveforms were then computed from epochs for each stimulus type (Fig. 1). Analysis of the selected parts of the major P100 was performed at the O1 and O2 electrode positions. Analysis of the main parts of the N170 was performed at P7 and P8 electrode positions. For N250, the analysis was performed at the F3, F4, C3, C4, FC3, and FC4 electrode positions.

### Statistical analysis

Statistical analyses were performed using SPSS 18.0 (IBM, Armonk, NY, USA). Data were presented as mean ± standard deviation (SD). Repeated measures analysis of variance (ANOVA) was used to test for differences in emotion recognition among the three groups at each intensity level for 3 (group) × 3 (emotion) × 3 (intensity) × 4 (position) repeated measures. ANOVA and the least significance difference (LSD) post hoc test were used to analyze the differences among the groups. A *P* value of <0.05 was considered to be significant.

## Results

### General information

Overall, a total of 86 participants were enrolled and completed the study. There were 30 cases of first-episode schizophrenia, 26 siblings of patients with schizophrenia, and 30 healthy volunteers. The demographic and clinical information for the schizophrenia, healthy control, and sibling groups have been previously published [13] (Additional file 1: Table S1). Eight of the patients with schizophrenia were treated with aripiprazole (maximum daily dose of 10 mg); ten were on olanzapine (maximum daily dose of 10 mg), and twelve were on risperidone (maximum daily dose of 4 mg). The duration of use of antipsychotic drugs did not exceed 2 weeks. The results of the facial expression recognition in this population have been previously presented [13] (Additional file 1: Table S2).

### Facial expression recognition

Overall, the mean reaction time required for facial expression recognition was similar among the groups (Table 1) for happiness, disgust, and fear, independent of intensity (low, moderate, vs. high) of the emotion. The significant differences in mean amplitudes and latencies for the P100, N170, and N250 components are presented in Table 2. The average P100 difference waveform for each group revealed significant differences among the groups, as well as significant interactions with lead position (F = 3.798, *P* = 0.002), with emotion and emotion intensity (F = 0.367, *P* = 0.008), and with emotion, emotion intensity, and lead position (F = 0.792, *P* = 0.003). These differences were mostly attributable to differences in amplitude between the healthy controls and the other two groups. As for P100 amplitudes, within the three groups, effects such as emotion, intensity, and position also showed significant differences (*P* < 0.05) (Table 2; please see the Additional Files for more details). However, no significant differences among the groups were observed for the N170 and N250 components.

**Table 1:** Comparison of the schizophrenia, healthy control, and sibling groups for recognition reaction time for three kinds of facial expression

	Healthy controls (*N* = 30)	Schizophrenia (*N* = 30)	Siblings (*N* = 26)	F	P
Happiness					
Low	616.4 ± 92.9	538.9 ± 99.9	614.3 ± 87.3	8.211	>0.05
Moderate	602.1 ± 94.9	493.9 ± 119.2	554.5 ± 64.7	6.611	>0.05
High	520.9 ± 81.9	459.1 ± 99.9	551.0 ± 69.6	1.134	>0.05
Disgust					
Low	600.4 ± 101.1	544.5 ± 125.7	608.9 ± 122.9	5.751	>0.05
Moderate	615.7 ± 76.4	531.7 ± 131.4	604.3 ± 98.3	7.167	>0.05
High	591.9 ± 102.1	528.8 ± 127.5	549.4 ± 111.0	2.036	>0.05
Fear					
Low	623.5 ± 98.5	509.1 ± 106.2	548.1 ± 98.9	10.631	>0.05
Moderate	642.0 ± 77.3	522.3 ± 111.0	591.6 ± 86.1	7.206	>0.05
High	594.9 ± 90.8	498.2 ± 118.9	548.0 ± 106.6	3.673	>0.05

Data are mean ± standard deviation

*P < 0.05 vs. the healthy control group

**Table 2:** P100, N170, and N250 amplitude and latency values that showed significant differences among groups

Emotion	Intensity	Site	Healthy controls	Schizophrenia	Sibling risk group	F	P
	P100 Amplitude						
Disgust	Low	O2	4.41 ± 2.93	3.25 ± 2.95	9.30 ± 2.40*^#^	20.498	<0.001
		P7	3.22 ± 2.54	2.86 ± 1.48	6.33 ± 2.46*^#^	12.903	<0.001
	Moderate	O2	4.76 ± 2.91	3.21 ± 3.68	10.56 ± 2.76*^#^	22.833	<0.001
		P7	3.20 ± 1.92	2.68 ± 1.85	6.39 ± 2.65*^#^	14.763	<0.001
		P8	5.33 ± 2.32	3.88 ± 2.95*	7.45 ± 3.33*^#^	6.882	0.002
	High	O2	4.58 ± 2.80	3.84 ± 3.42	9.94 ± 2.35*^#^	18.900	<0.001
		P7	3.21 ± 2.49	2.93 ± 1.73	6.22 ± 2.99*^#^	9.772	<0.001
		P8	5.77 ± 2.47	4.30 ± 3.16*	7.96 ± 3.21*^#^	6.698	0.002
Happiness	Low	O2	4.16 ± 2.78	3.73 ± 2.89	8.72 ± 2.10*^#^	15.030	<0.001
		P7	2.97 ± 2.50	3.08 ± 1.79	5.60 ± 2.74*^#^	6.106	0.004
	Moderate	O2	4.26 ± 3.13	3.59 ± 2.65	8.69 ± 2.85*^#^	14.624	<0.001
		P7	3.37 ± 2.53	2.75 ± 1.87	5.97 ± 2.54*^#^	9.143	<0.001
		P8	5.26 ± 2.79	4.36 ± 2.77	6.96 ± 3.26^#^	3.502	0.037
	High	O2	4.57 ± 2.88	4.51 ± 2.79	10.54 ± 2.23*^#^	24.627	<0.001
		P7	3.85 ± 2.57	3.04 ± 1.52	7.11 ± 1.86*^#^	18.537	<0.001
Fear	Low	O2	4.25 ± 2.37	4.00 ± 2.28	9.68 ± 2.08*^#^	30.456	<0.001
		P7	2.70 ± 2.51	2.98 ± 1.80	6.95 ± 2.08*^#^	18.939	<0.001
		P8	4.56 ± 2.64	4.13 ± 2.45	7.02 ± 2.89*^#^	5.559	0.006
	Moderate	O1	3.88 ± 3.05	3.54 ± 2.16	5.93 ± 1.84*^#^	4.427	0.017
		O2	4.29 ± 2.52	3.75 ± 2.69	8.27 ± 1.77*^#^	15.671	<0.001
		P7	2.99 ± 2.53	2.54 ± 1.31	5.37 ± 2.61*^#^	8.352	0.001
	High	O2	4.70 ± 2.53	4.25 ± 2.57	10.09 ± 2.12*^#^	26.673	<0.001
		P7	3.54 ± 2.38	3.11 ± 1.57	6.47 ± 2.19*^#^	12.968	<0.001
	P100 latency						
Disgust	Moderate	O1	119.50 ± 16.85	119.26 ± 22.60	90.85 ± 23.76*^#^	9.061	<0.001
		O2	120.11 ± 19.25	102.63 ± 29.21*	115.46 ± 16.50	3.156	0.050
	High	O1	116.00 ± 17.22	115.04 ± 23.17	80.31 ± 30.78*^#^	11.269	<0.001
Happiness	High	O1	118.44 ± 19.64	119.08 ± 22.17	99.92 ± 23.21*^#^	3.808	0.028
Fear	Moderate	O1	111.83 ± 24.34	122.31 ± 20.56	103.92 ± 22.42^#^	3.201	0.049
	High	O1	121.89 ± 19.12	114.92 ± 28.07	92.08 ± 26.18*^#^	5.650	0.006
	N170 Amplitude						
Happy	Low	O2	2.17 ± 2.56	1.92 ± 2.74	4.73 ± 3.58*^#^	4.216	0.020
	N170 latency						
Disgust	Low	P8	171.95 ± 15.96	185.11 ± 16.45*	170.54 ± 14.04^#^	5.958	0.005
	Moderate	P8	173.39 ± 11.58	185.37 ± 16.38*	171.69 ± 11.54^#^	5.947	0.005
	High	P8	176.50 ± 10.93	184.26 ± 13.14*	172.23 ± 10.56^#^	5.088	0.009
Happiness	Moderate	O1	178.89 ± 15.95	184.54 ± 20.53	196.46 ± 17.94*	3.422	0.040
		P8	175.00 ± 11.55	186.69 ± 16.85*	172.85 ± 12.23^#^	5.513	0.007
	High	P7	177.28 ± 16.62	179.72 ± 24.47	159.46 ± 23.19*^#^	3.899	0.026
		P8	173.44 ± 15.86	185.56 ± 18.27*	174.46 ± 11.94	3.596	0.034
Fear	Moderate	O1	179.61 ± 14.46	174.27 ± 25.56	199.00 ± 20.92*^#^	5.804	0.005
		P8	174.72 ± 12.43	182.27 ± 14.42*	170.62 ± 11.46^#^	3.855	0.027
	High	O2	173.17 ± 17.05	189.73 ± 20.45*	170.62 ± 13.26^#^	6.855	0.002
		P8	175.28 ± 13.06	186.92 ± 15.08*	171.38 ± 12.93^#^	6.649	0.003
	N250 Amplitude						
Fear	High	F3	2.18 ± 1.65	2.26 ± 1.21	3.87 ± 1.71*^#^	6.307	0.003
	N250 latency						
Happiness	Low	F3	271.62 ± 59.72	253.35 ± 53.38	318.92 ± 29.66*^#^	5.481	0.007
	Moderate	F3	268.05 ± 56.72	261.13 ± 58.93	315.00 ± 40.72*^#^	4.308	0.018
		F4	257.29 ± 43.70	262.70 ± 47.27	308.85 ± 38.41*^#^	7.115	0.002
	High	F4	252.71 ± 49.28	254.95 ± 55.33	314.15 ± 24.42*^#^	8.153	0.001
Disgust	Low	F3	280.57 ± 47.55	286.52 ± 43.02	319.46 ± 28.48*^#^	3.915	0.026
	Moderate	F3	278.27 ± 43.37	266.04 ± 51.72	313.15 ± 40.15*^#^	3.981	0.024
	High	F4	251.59 ± 49.60	256.22 ± 56.27	304.77 ± 42.57*^#^	4.999	0.010
Fear	Low	F4	277.00 ± 42.43	250.78 ± 53.06	301.54 ± 35.87^#^	3.920	0.026
		FC3	274.14 ± 47.56	253.09 ± 53.71	312.23 ± 43.91*^#^	4.746	0.013
	Moderate	F3	265.10 ± 45.55	279.30 ± 49.57	313.38 ± 38.47*^#^	4.585	0.014
	High	F3	274.67 ± 50.85	269.70 ± 56.07	313.92 ± 41.08*^#^	3.627	0.033
		FC3	271.90 ± 52.56	263.41 ± 61.82	316.85 ± 32.06*^#^	4.774	0.012

Data are presented as mean ± standard deviation. Because of the large amount of data only the results with statistical differences are presented in this table

*P < 0.05 vs. the control group

^#^ *P* < 0.05 vs. the schizophrenia group

All ANOVA analyses were performed with 2° of freedom

## Discussion

The aim of this study was to investigate the extent to which facial emotion recognition was impaired in patients with schizophrenia and whether similar or more subtle impairments in siblings could also be identified using ERP. The results showed that there were significant differences among patients with schizophrenia, siblings, and healthy controls for P100 amplitude, position, emotion, and intensity. Post hoc analysis showed significantly larger amplitudes in the schizophrenia group at O1, O2, P7, and P8. These differences were not attributable to significant differences between the siblings and healthy controls, suggesting that possible early or low-grade facial emotion recognition impairment in the siblings is not evident in the EEG data.

We investigated facial emotion recognition in patients with first-episode schizophrenia, their siblings, and healthy controls, and found that patients with schizophrenia performed worse overall when compared to the other groups. This is relatively consistent with our previous study that looked at the relationship of facial emotion recognition with executive functions [13], as well as with prior studies of emotional face recognition in schizophrenia [23]. Our study enrolled patients with first-episode schizophrenia, which minimized the long-term side effects of the illness and medication, and suggests that these changes may be part of the disease rather than associated with chronicity or treatment. The expression of facial emotion is a complex social cognition ability, which involves several stages for successful processing, including initial visual processing, structural encoding of a face and later association of the representation with cognitive, semantic, and affective information for distinguishing between the emotions [24, 25]. Our findings suggested a generalized form of cognitive impairment and structural or attentional encoding deficits in schizophrenia.

An important aspect of our study was inclusion of siblings of patients with schizophrenia as a high-risk group. Overall, siblings were less able to correctly recognize three kinds of facial expression compared to the healthy controls, but this difference was not statistically significant. Ibanez et al. also found that the accuracy of facial expression recognition showed a poorer, but non-significant performance in relatives of patients with schizophrenia compared to controls [26]. The siblings, who are at higher risk of developing schizophrenia, may have some degree of impairment in facial expression recognition but the ethology results may be harder to detect. Indeed, the laboratory setting and the measurement devices are factors that render the environment unnatural for these patients, masking some of the effects. Despite normal N170 and N250 amplitude and latency among the three groups in our study, previous research has found that patients with schizophrenia showed significantly longer latency compared to healthy controls [27]. These differences may be due to the small sample size of our study, or reflect the fact that the patients with schizophrenia in our study were very recently diagnosed and so are likely to be in the early stage of the disease. This might suggest that facial recognition is a gradual conversion process from quantitative to qualitative information.

This study has some limitations. The sample size was small because. Despite the large number of patients treated within the department, the number of patients with siblings was relatively small. Another limitation of the study is likely to be the medication received by the patients. Upon diagnosis, each patient received antipsychotic medication with aripiprazole, olanzapine, or risperidone. Despite the fact the patients had received their medication for less than 2 weeks, the doses varied among patients. This may have had an influence on the results but we did not assess whether the results were influenced by the drug received or by the dose.

## Conclusions

In conclusion, patients with schizophrenia, siblings, and healthy controls showed significant differences in amplitude of P100, electrode position, emotion, and intensity. While there were significantly larger amplitudes in the schizophrenia group at O1, O2, P7, and P8, these differences were not significant between the siblings and healthy controls, suggesting that possible early or low-grade facial emotion recognition impairment in siblings is not evident in the EEG data. Thus, it cannot be used for early diagnosis of siblings. This result may be due to the small sample size and while ERP anomalies among patients with first-episode schizophrenia, siblings, and healthy controls may provide information for the early diagnosis of schizophrenia, as an endophenotype of schizophrenia, this requires further study.

## Additional file

Additional file 1: Table S1. Demographic and clinical information for the study participants (mean ± SD). **Table S2.** Comparison of the schizophrenia, healthy controls and high-risk groups for correct recognition of three kinds of facial expression. (DOC 52 kb)

## Abbreviations

ANOVA: Analysis of variance
DSM-IV: Statistical Manual of Mental Disorders, 4th edition
EASY: Early Assessment service for Young People with Psychosis
EEG: Electroencephalography
ERP: Event-related potential
N170: Negative potential 170 ms after stimulus onset
N250: Peak generated at fronto-central sites
P100: Positive potential about 100 ms after stimulus onset
PANSS: Positive and Negative Symptom Scale
SCID: Structured Clinical Interview for Diagnostic
SD: Standard deviation
