# Supplementary material for: Changes in event-related potentials in patients with first-episode schizophrenia and their siblings
Source: BMC Psychiatry. 2017 Jan 17;17:20. doi: 10.1186/s12888-016-1189-7 (PMC5240372; doi:10.1186/s12888-016-1189-7)
Supplement: Additional file 1: Table S1. — Demographic and clinical information for the study participants (mean ± SD). Table S2. Comparison of the schizophrenia, healthy controls and high-risk groups for correct recognition of three kinds of facial expression. (DOC 52 kb) [file 12888_2016_1189_MOESM1_ESM.doc]

**Additional file 1**

**Table S1.** Demographic and clinical information for the study participants (mean ±SD)

|  | **Schizophrenia**  **(N=30)** | **Heathy controls**  **(N=30)** | **High risk**  **(N=26)** | **P** |
| --- | --- | --- | --- | --- |
| **Age(years)** | 22.28±3.17 | 24.63±2.19 | 23.87±1.59 | ＞0.05 |
| **Education(years)** | 11.95±3.17 | 13.17±3.52 | 13.92±1.83 | ＞0.05 |
| **Male/Female(n)** | 15/15 | 15/15 | 12/14 | ＞0.05 |
| **PANSS** |  |  |  |  |
| **Positive** | 28.4±6.5 |  |  |  |
| **Negative** | 23.7±7.9 |  |  |  |
| **General** | 38±8.6 |  |  |  |

**Table S2.** Comparison of the schizophrenia, healthy controls and high-risk group for correct recognition of three kinds of facial expression

|  | **Schizophrenia**  **(N=30)** | **Heathy controls**  **(N=30)** | **High risk**  **(N=26)** | **F** | **P** |
| --- | --- | --- | --- | --- | --- |
| **Happy** |  |  |  |  |  |
| **Low** | 21.0±13.7 | 26.0±9.7 | 29.0±14.5 | 0.885 | ＞0.05 |
| **Moderate** | 57.0±24.9 | 83.0±12.1 | 82.0±14.0 | 13.129 | ＜0.01 |
| **High** | 70.0±27.9 | 95.0±5.7 | 93.0±8.2 | 12.068 | ＜0.01 |
| **Disgust** |  |  |  |  |  |
| **Low** | 25.0±20.3 | 11.0±9.0 | 25.0±18.4 | 5.225 | ＜0.01 |
| **Moderate** | 43.0±21.4 | 68.0±21.2 | 73.0±15.5 | 11.037 | ＜0.01 |
| **High** | 61.0±28.4 | 84.0±22.3 | 87.0±15.7 | 6.110 | ＜0.01 |
| **Fear** |  |  |  |  |  |
| **Low** | 20.0±14.0 | 16.0±11.4 | 14.0±10.0 | 0.853 | ＞0.05 |
| **Moderate** | 45.0±19.2 | 74.0±13.3 | 69.0±16.1 | 18.547 | ＜0.01 |
| **High** | 60.0±26.6 | 88.0±9.0 | 82.0±14.8 | 13.409 | ＜0.01 |
